# Low prevalence of equine coronavirus in foals in the largest thoroughbred horse breeding region of Japan, 2012–2014

**DOI:** 10.1186/s13028-015-0149-4

**Published:** 2015-09-22

**Authors:** Manabu Nemoto, Yasuhiro Oue, Tohru Higuchi, Yuta Kinoshita, Hiroshi Bannai, Koji Tsujimura, Takashi Yamanaka, Takashi Kondo

**Affiliations:** Epizootic Research Center, Equine Research Institute, Japan Racing Association, 1400-4 Shiba, Shimotsuke, Tochigi 329-0412 Japan; Hokkaido Kushiro Livestock Hygiene Service Center, 127-1 Otanoshike, Kushiro, Hokkaido 084-0917 Japan; Hidaka Agriculture Mutual Aid Association, 200 Higashihourai, Mitsuishi, Shinhidaka-cho, Hidaka-gun, Hokkaido, 059-3105 Japan

**Keywords:** Equine coronavirus, Foal, Japan, Reverse transcription loop-mediated isothermal amplification, Real-time reverse transcription-polymerase chain reaction

## Abstract

**Background:**

Equine coronavirus (ECoV) is considered to be a diarrheic pathogen in foals. In central Kentucky in the United States, it has been shown that approximately 30 % of thoroughbred foals are infected with ECoV and thus it is considered widely prevalent. In contrast, the epidemiology of ECoV and its relationship to diarrhea in foals are poorly understood in Japan. We investigated ECoV in rectal swabs collected from thoroughbred foals in Japan.

**Results:**

We collected 337 rectal swabs from 307 diarrheic foals in the Hidaka district of Hokkaido, the largest thoroughbred horse breeding region in Japan, between 2012 and 2014. In addition, 120 rectal swabs were collected from 120 healthy foals in 2012. These samples were tested by reverse transcription loop-mediated isothermal amplification and a real-time reverse transcription-polymerase chain reaction. All samples collected from diarrheic foals were negative, and only three samples (2.5 %) collected from healthy foals were positive for ECoV. Compared with central Kentucky, ECoV is not prevalent among thoroughbred foals in the Hidaka district of Hokkaido.

**Conclusion:**

ECoV is not prevalent and was not related to diarrhea in thoroughbred foals in the Hidaka district of Hokkaido between 2012 and 2014.

## Findings

Certain pathogens are common causes of diarrhea in foals, equine rotavirus being the most common [1]. Equine coronavirus (ECoV) is also a diarrheic pathogen in foals [2]. Recently, several ECoV outbreaks in adult horses occurred in the United States [3] and Japan [4–6]. Fever, anorexia, lethargy, leukopenia and digestive disorders were observed, and these clinical signs were reproduced in an experimental challenge study [7]. Equine coronavirus was also detected in fecal samples of diarrheic foals in the United States [8, 9], but there have been no reports of an ECoV outbreak in foals.

Slovis et al. [10] reported that approximately 30 % of healthy and diarrheic thoroughbred foals in central Kentucky in the United States were infected with ECoV, using a real-time reverse transcription-polymerase chain reaction (RT-PCR) assay. These results indicate that ECoV is prevalent among thoroughbred foals in central Kentucky. In Japan, ECoV outbreaks had previously occurred only in the draft racehorse population. Draft racehorses include French Percheron, Breton and Belgian horses, with body weights around 1000 kg. Outbreaks of ECoV had not been reported in the Japanese thoroughbred horse population, and its epidemiology is poorly understood. It is also unclear whether ECoV is related to diarrhea in Japanese foals. Therefore, we investigated ECoV using molecular diagnostic methods on rectal swabs collected from thoroughbred foals in the Hidaka district of Hokkaido, which is the largest thoroughbred horse breeding region in Japan.

Between 2012 and 2014, we collected 337 rectal swabs from 307 diarrheic foals aged 2 days to 5 months in the Hidaka district of Hokkaido by using BD BBL CultureSwab EZ (Becton, Dickinson and Company, Fukushima, Japan). Rectal swabs of twenty-two and four diarrheic foals were collected twice and three times, respectively. Multiple samples were collected with 3–106 days intervals (average 28.5 days). We collected 121 samples from 63 farms in 2012, 121 samples from 63 farms in 2013 and 95 samples from 44 farms in 2014. Rectal swabs from diarrheic foals were stored at −20 °C in the veterinary clinic until transport. They were transported to the diagnostic laboratory at around −18 °C, and after arrival they were kept at −80 °C until use. Additionally, 120 rectal swabs were collected from 120 healthy foals on 30 farms in the same region in 2012 by using BD BBL CultureSwab Plus (Becton, Dickinson and Company). Healthy foals were between 1 and 80 days of age. Rectal swabs from healthy foals were stored and transported at 4 °C for several days. After arrival, they were kept at −80 °C until use. These samples were immersed in maintenance medium [11] or phosphate buffered saline. Viral RNA was extracted from samples with MagNA Pure LC Total Nucleic Acid Isolation Kit (Roche Diagnostics, Mannheim, Germany).

Reverse transcription loop-mediated isothermal amplification (RT-LAMP) and real-time RT-PCR assays were selected because these molecular methods have high sensitivity for ECoV [12]. The RT-LAMP reaction was performed using a primer set described previously [12] and a Loopamp RNA Amplification Kit (Eiken Chemical, Tokyo, Japan) according to the manufacturer’s instructions. Calcein, a fluorescent detection reagent (Eiken Chemical, Tokyo, Japan), was added to the reaction mixture for visual detection. The mixtures were incubated at 60 °C for 40 min and then heated at 95 °C for 2 min to terminate the reaction.

Real-time RT-PCR assay was conducted using a primer set described previously (ECoV-380f, ECoV-522r and ECoV-436p) [3] and TaqMan Fast Virus 1-Step Master Mix (Applied Biosystems, Foster City, CA, USA) according to the manufacturer’s instructions. Thermal cycling was performed as [7]: 50 °C for 5 min and 95 °C for 20 s, followed by 40 cycles at 94 °C for 3 s and 60 °C for 30 s.

For sequence and phylogenetic analysis of the nucleocapsid (N) gene, RT-PCR was performed using the primer set ECoV-Nf (5′-tcaggcatggacaccgcattgtt-3′) and ECoV-Nr (5′-ccaggtgccgacataaggttcat-3′) [5] using PrimeScript II High Fidelity One Step RT-PCR Kit (Takara Bio, Otsu, Japan). RT-PCR products were directly sequenced commercially by Fasmac (Atsugi, Japan). Sequence analysis was performed using the BLAST and CLUSTALW programs, and Vector NTI Advance 11 software (Invitrogen, Carlsbad, CA, USA). Phylogenetic analysis of nucleotide sequences was conducted with MEGA software Version 5.2 [13]. A phylogenetic tree was constructed based on nucleotide sequences using the neighbor-joining method. Statistical analysis of the tree was performed with the bootstrap test (1000 replicates) for multiple alignments. The nucleotide sequences of the N gene in Hidaka-No.61/2012 and Hidaka-No.119/2012 have been deposited in GenBank/EMBL/DDBJ under the accession numbers LC054263 and LC054264, respectively. The N gene of NC99 (AF251144), Obihiro2004 (AB671298), Tokachi09 (AB555559) and Obihiro12-1 (AB775893) were used to compare with that of Hidaka-No.61/2012 and Hidaka-No.119/2012.

All 337 samples collected from diarrheic foals were negative for ECoV on RT-LAMP and real-time RT-PCR assays. Three samples collected from healthy foals aged 30–39 days at two farms were positive for ECoV on RT-LAMP and real-time RT-PCR assays. N gene amplification was attempted in the three positive samples and was successful in two of them (Hidaka-No.61/2012 collected at A farm in April 2012 and Hidaka-No.119/2012 collected at B farm in April 2012).

Sequence analysis was performed for the N gene (1341 nucleotides and 446 amino acids) of Hidaka-No.61/2012 and Hidaka-No.119/2012. The nucleotide and amino acid sequence identities between the two samples analyzed in this study and in four other ECoVs (NC99 [9, 14], Obihiro2004 [4], Tokachi09 [5] and Obihiro12-1 [6] strains) are shown in Table 1. The sequence identity of Hidaka-No.61/2012 with the other four ECoVs was 98.2–99.9 % at the nucleotides and 97.3–100 % at the amino acids. Sequence identity of Hidaka-No.119/2012 with the other four ECoVs was 97.8–99.2 % at the nucleotides and 96.6–98.9 % at the amino acids.

**Table 1:** Sequence identities of the nucleocapsid gene between ECoVs identified in this study and other ECoVs

Strains	Hidaka-No.61/2012		Hidaka-No.119/2012	
	Nucleotide (nt)	Amino acid (aa)	Nucleotide	Amino acid
NC99	98.2 % (1317/1341nt)	97.3 % (434/446aa)	97.8 % (1311/1341nt)	96.6 % (431/446aa)
Obihiro2004	98.5 % (1321/1341nt)	97.5 % (435/446aa)	98.2 % (1317/1341nt)	97.1 % (433/446aa)
Tokachi09	99.3 % (1331/1341nt)	98.9 % (441/446aa)	99.1 % (1329/1341nt)	98.7 % (440/446aa)
Obihiro12-1	99.9 % (1340/1341nt)	100 % (446/446aa)	99.2 % (1330/1341nt)	98.9 % (441/446aa)

**Fig. 1:**
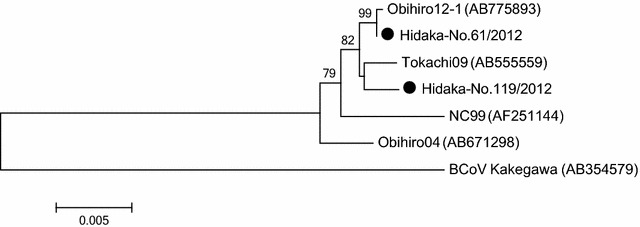
Phylogenetic analysis of the nucleotide sequences of the nucleocapsid gene of equine coronavirus. *Closed circles* indicate two equine coronaviruses analyzed in this study (Hidaka-No.61/2012 and Hidaka-No.119/2012). The percentage bootstrap support is indicated by the value at each node; values <70 % are omitted. *Scale bars* indicate nucleotide substitutions per site. BCoV (Bovine coronavirus) Kakegawa strain is used as an out-group

Phylogenetic analysis was performed for the nucleotide sequences of the N gene (Fig. 1). Phylogenetic analysis showed that Hidaka-No.61/2012 and Hidaka-No.119/2012 are closely related to the Obihiro12-1 and Tokachi09 strains, respectively.

In this study, all diarrheic samples were negative for ECoV. This indicates that ECoV is not a causative agent of diarrhea in thoroughbred foals in Hidaka district of Hokkaido. Using 120 rectal swabs collected from healthy foals, only three samples (2.5 %) were positive for ECoV. Rectal swabs from healthy foals preserved at worse condition than that of diarrheic foals as described above. However, ECoV was only detected in rectal swabs from healthy foals, and therefore it is unclear whether storage condition influenced the result in this study. Compared with central Kentucky, ECoV is not prevalent among thoroughbred foals in Hidaka district of Hokkaido, but some outbreaks have occurred in draft racehorses. One reason for the lack of ECoV positive samples in thoroughbred foals may be that they do not usually have contact with draft horses. These results suggest that the prevalence of ECoV varies greatly depending on region. To further show this, it is necessary to investigate ECoV in other countries as well.

In sequence and phylogenetic analyses of the N gene, there is little difference between Hidaka-No.61/2012 and Obihiro12-1. Obihiro12-1 was isolated in the ECoV outbreak in March 2012 [6], and therefore the Hidaka-No.61/2012 collected in April 2012 may have come from a virus circulating during the outbreak. In contrast, Hidaka-No.119/2012 did not come from an Obihiro12-1-like virus based on sequence and phylogenetic analyses. Ultimately, Hidaka-No.61/2012- and Hidaka-No.119/2012-like viruses did not establish themselves in thoroughbred foals in Hidaka district of Hokkaido, because ECoV was not detected in any diarrheic samples in 2013 or 2014.

In conclusion, this study shows that ECoV is not prevalent and was not related to diarrhea in thoroughbred foals in the Hidaka district of Hokkaido between 2012 and 2014.
